# Predicting COVID-19 Cases in South Korea Using Stringency and Niño Sea Surface Temperature Indices

**DOI:** 10.3389/fpubh.2022.871354

**Published:** 2022-06-03

**Authors:** Imee V. Necesito, John Mark S. Velasco, Jaewon Jung, Young Hye Bae, Younghoon Yoo, Soojun Kim, Hung Soo Kim

**Affiliations:** ^1^Department of Civil Engineering, Inha University, Incheon, South Korea; ^2^Department of Clinical Epidemiology, College of Medicine, University of the Philippines, Manila, Philippines; ^3^Institute of Molecular Biology and Biotechnology, National Institutes of Health, University of the Philippines, Manila, Philippines; ^4^Department of Hydro Science and Engineering Research, Korea Institute of Civil Engineering and Building Technology, Gyeonggi-do, South Korea

**Keywords:** COVID-19, stringency index, Niño SST index, NARX, South Korea

## Abstract

Most coronavirus disease 2019 (COVID-19) models use a combination of agent-based and equation-based models with only a few incorporating environmental factors in their prediction models. Many studies have shown that human and environmental factors play huge roles in disease transmission and spread, but few have combined the use of both factors, especially for SARS-CoV-2. In this study, both man-made policies (Stringency Index) and environment variables (Niño SST Index) were combined to predict the number of COVID-19 cases in South Korea. The performance indicators showed satisfactory results in modeling COVID-19 cases using the Non-linear Autoregressive Exogenous Model (NARX) as the modeling method, and Stringency Index (SI) and Niño Sea Surface Temperature (SST) as model variables. In this study, we showed that the accuracy of SARS-CoV-2 transmission forecasts may be further improved by incorporating both the Niño SST and SI variables and combining these variables with NARX may outperform other models. Future forecasting work by modelers should consider including climate or environmental variables (i.e., Niño SST) to enhance the prediction of transmission and spread of severe acute respiratory syndrome coronavirus 2 (SARS-CoV-2).

## Introduction

The SARS-CoV-2 virus, which is the causative agent of the coronavirus disease (COVID-19) was first reported in Wuhan, China in December 2019 (1). Since then, COVID-19 has been declared a pandemic and has become a global public health threat for almost 2 years (2). Closure of borders, nationwide lockdowns, and reduced air flights were the countermeasures imposed by different countries to contain the spread of the disease (3–6). In South Korea, the first case of COVID-19 was detected on 20 January 2020 and as of 28 February 2022 there have been 2,665,077 confirmed COVID-19 cases and 7,783 deaths recorded (https://covid19.who.int/region/wpro/country/kr). Various mathematical models have been used to forecast the transmission and spread of COVID-19 (7–9), with different kinds of models associated with specific strengths and weaknesses. While most COVID-19 models use a combination of agent-based and equation-based (i.e., SIR, SEIR) models (2, 10), the main objective of obtaining a prediction model sufficiently accurate to be able to plan and target effective and optimal countermeasures (11), which will subsequently help in decreasing the number of cases, minimizing the number of deaths, and limiting impact to a country's economy (11), only a few studies have incorporated environmental factors in their prediction models (12).

Understanding how climate variability can affect infectious disease transmission is important. Various climatic factors (i.e., temperature, precipitation, humidity, etc.) have been implicated to have complex effects on the disease dynamics of many water-borne and vector-borne infectious diseases (13). Epidemics of dengue fever and malaria have been linked to the El Niño Southern Oscillation (ENSO) phenomenon (14, 15). The increasing intensity of certain diseases of public health concern (i.e., chikungunya, hantavirus, Rift Valley fever, cholera, plague, and Zika) was also significantly associated with ENSO-induced climate anomalies (16). Some studies have emphasized that sea surface temperature (SST) plays a key role in the occurrence of weather systems (17, 18), which are both dependent on the fluctuations of both atmosphere and sea (19), while Byrne and O'Gorman (20) showed how temperature and humidity have changed due to the warming of the oceans. Some studies have pointed out that temperatures, wind speed, and humidity may play major roles in COVID-19 prediction modeling (21–23). Stringency policies were also used by Soobhug et al. (24), as one of the governing factors in predicting COVID-19 in Mauritius.

SARS-CoV-2 is an enveloped RNA virus, structurally similar to other RNA viruses (i.e., Middle East respiratory syndrome-related coronavirus and HcoV-NL63 human coronavirus) (25), which display seasonal dynamics due to their physical properties. The role of climate, higher temperatures, more intense UV radiation during summer, high humidity and precipitation, and their effects on the transmission of SARS-CoV-2 have been discussed in several publications (12, 26–33). Should COVID-19 persist endemically and continue in the long term, it is important to determine whether COVID-19 will follow seasonally-driven patterns of infection or whether COVID-19 transmission dynamics and outbreaks will be potentially affected by short- and long-term climate changes (34).

Lee et al. (35) made a comparative analysis of COVID-19 epidemic transmission in China, Japan, Thailand, Taiwan, Malaysia, Singapore, Germany, France, Canada, the UK, and South Korea by incorporating each country's reproduction numbers and prevention and control measures. In the study, it was concluded that South Korea's high detection rate through massive testing has been the key factor in its success to contain the outbreak of the virus. On the other hand, through the comparative analyses made by Chen et al. (36) of the four East Asian countries (China, Japan, Singapore, and South Korea), it was concluded that the containment strategy of South Korea (together with China and Singapore), which include a rapid National Emergency response system, border control measures, screening, and testing measures, and massive public health and social distancing measures have been the key to its success of slowing down the epidemic. In the study of Chen et al. (36), it was also emphasized that the mitigation strategy, which was typically done by most countries, is just secondary compared to a containment strategy.

The accuracy of assumptions in the field of epidemiology can be best represented by mathematical representations. Some papers have examined transmission modeling of COVID-19 using different approaches such as path analysis (37), fractional differential equations (38), spatial autocorrelation, hot spot, and Spatio-temporal scanning statistics (39). However, disease-modeling using deep learning has been proven to help in outlining disease progression and spread (40–42), predict trends (43, 44) and risks (45), and even help the governments in decision-making (46, 47). NARX is a type of deep learning, which makes use of the past values of a time series, alongside the current and past values of the exogenous variables. This deep learning method has been used by several researchers to predict disease incidence and impacts in countries located in regions like Asia (48, 49), Europe, Middle East (50), and the Americas (51). Unlike other deep learning methods, NARX has an advantage by integrating multiple variable inputs with the autoregressive inputs, which helps in increasing the accuracy of results (52). Among these studies, NARX has shown promising qualities for disease modeling applications. This paper intends to propose and utilize the novel combination of Niño SST indices and the Stringency Index (SI) with the Non-linear Autoregressive Exogenous (NARX) model to predict COVID-19 incidence in South Korea.

## Materials and Methods

### Data

Data were gathered from a publicly available database (www.ourworldindata.org) maintained by the Oxford Coronavirus Government Response Tracker (OxCGRT). The COVID-19 daily data of South Korea from January 21, 2020 to December 31, 2020, was used in this study with January 21 to September 30, 2020, as the training data and October 1 to December 31, 2020, as the testing data.

**Figure 1:**
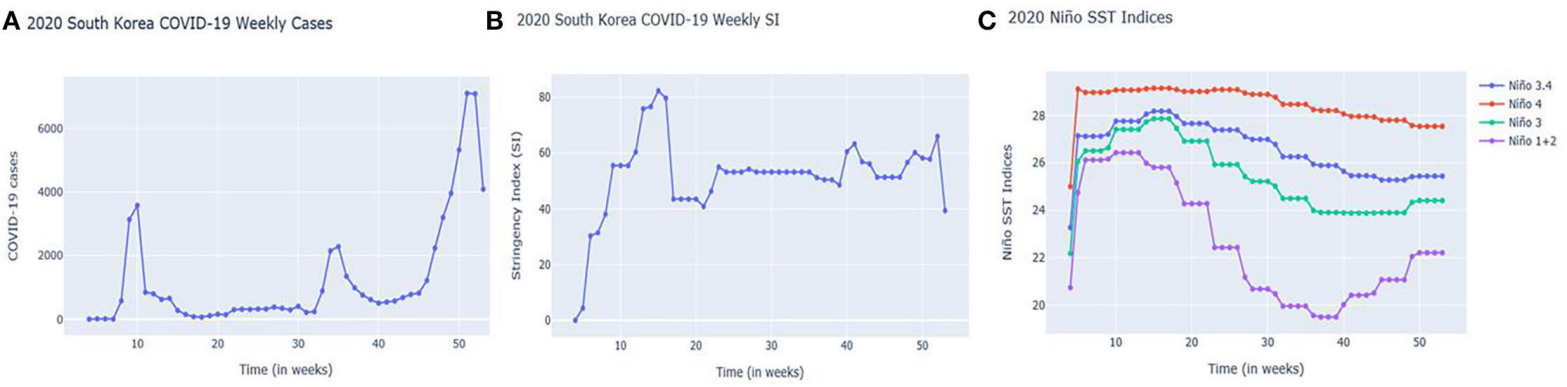
South Korea's Weekly **(A)** COVID-19 cases; **(B)** Stringency Index (SI); and **(C)** Niño SST indices from January 21, 2020 to December 31, 2020 (4th week to 53rd week).

South Korea first recorded its COVID-19 case in January 2020. The initial response with information technology supplemented the contract tracing and flattening of the first wave of the COVID-19 case curve. However, thousands of cases were still eventually recorded. In fact, 3,578 cases were recorded during the first 10 weeks followed by 2,282 cases by week 35 (August), and by the end of 2020, 7,107 weekly cases were recorded (See Figure 1A).

South Korean government also implemented COVID-19 regulations as represented by Stringency Index (SI) (See Figure 1B). As the case numbers increased, regulations as reflected by SIs also increased during weeks 6–10 (February to March) and continued until weeks 15–16 (April). Due to the low number of cases during summer 2020 (200–400 cases weekly), SIs also decreased. There were spikes of cases in the 34–35th week (August to September) (probably due to holidays) followed by an increasing trend of cases in the 40th week (October) onwards. SIs have shown an increasing-decreasing pattern, potentially attributed to economic factors as the government tried to reopen businesses (https://crisis24.garda.com/alerts/2020/10/south-korea-authorities-to-ease-some-COVID-19-restrictions-from-october-12-update-37).

Looking at the Niño SST indices, it is clear that Niño1+2 in Figure 1C is more prone to abrupt changes. A sudden monthly drop can be noticed starting in week 14 (March to April) onwards but increased as it reached the year-end weeks: 40 (October), 45 (November), and 50 (December). Niño1+2 indices were also found to be influential in the monthly precipitation of South Korea based on the study done by Kim et al. (53).

The National Water Service–Climate Prediction Center (https://origin.cpc.ncep.noaa.gov) uses overlapping seasons which are divided as DJF (Dec-Jan-Feb), JFM (Jan-Feb-Mar), FMA (Feb-Mar-Apr), MAM (Mar-Apr-May), AMJ (Apr-May-Jun), MJJ (May-Jun-Jul), JJA (Jun-Jul-Aug), JAS (Jul-Aug-Sep), ASO (Aug-Sep-Oct), SON (Sep-Oct-Nov), OND (Oct-Nov-Dec), and NDJ (Nov-Dec-Jan). Thus, due to the seasonal nature of Niño SST indices, the authors used OND (Oct-Nov-Dec, October 1 to December 31, 2020) as part of the testing data. As previously stated, the training data used in this study is from January 21 to September 30, 2020. Also, due to the potential COVID-19 case count interference from the administration of COVID-19 vaccines, the 2021 COVID-19 dataset was not used. To directly compare the performance of our model with another COVID-19 published model, we used another set of training and testing dates which were similar to the dates used by the model of Kafieh et al. (54). The authors used S. Korea COVID-19 cases from January 22 to July 30, 2020, for the training data and COVID-19 cases from August 1–31, 2020, for the testing data. Prediction data were set from Sept 1 to October 12, 2020. The descriptive statistics of the data used in this study can be summarized in Table 1A.

**Table 1A:** Descriptive statistics of the data used (January 21, 2020 to December 31, 2020).

	**SI**	**Cases**	**Niño3.4**	**Niño4**	**Niño3**	**Niño1+2**
Count	346	346	346	346	346	346
Mean	52.29	178.52	26.69	28.58	25.50	22.66
Std	14.39	261.80	0.99	0.57	1.41	2.46
Min	0	0	25.28	27.54	23.88	19.5
Max	82.41	1,237.0	28.18	29.17	27.86	26.43

### Sea Surface Temperature

According to the US Environmental Protection Agency (55) (https://www.epa.gov/climate-indicators/climate-change-indicators-sea-surface-temperature), SST stands for “Sea Surface Temperature” and is defined as the temperature of the surface of the ocean water. In the tropical Pacific, indices are used to monitor the temperature based on the average anomalies. As defined by the National Oceanic and Atmospheric Administration, (56), during La Niña events, the trade winds are stronger, which can push more warm water toward Asia with increased upwelling (rising of cold water) to the west coast of the Americas, bringing cold water to the surface. On the other hand, El Niño events have weak trade winds where warm water is pushed back east toward the west coast of the Americas.

The following is the definition of the Niño Indices and their corresponding regions (longitude, latitude) based on the Climate Data Guide of the National Center for Atmospheric Research (NCAR) (57).

Niño 1+2 (0-10S, 90W-80W): The smallest and eastern-most of the Niño SST regions, which encompasses the region of coastal South America where El Niño, was first recognized by the local populations.

Niño 3 (5N-5S, 150W-90W): This region was once the primary focus for monitoring and predicting El Niño. However, it was later found out that Niño 3.4 and ONI are better suited for defining El Niño and La Niña events.

Niño 3.4 (5N-5S, 170W-120W): its anomalies correspond to the average equatorial SSTs across the Pacific from the dateline to the South American coast. When the Niño 3.4 SSTs exceed +/- 0.4 C for 6 months or greater, El Niño or La Niña events are said to occur.

Niño 4 (5N-5S, 160E-150W): its anomalies correspond to the central equatorial Pacific. This region appears to have less variance than the other Niño regions.

**Figure 2:**
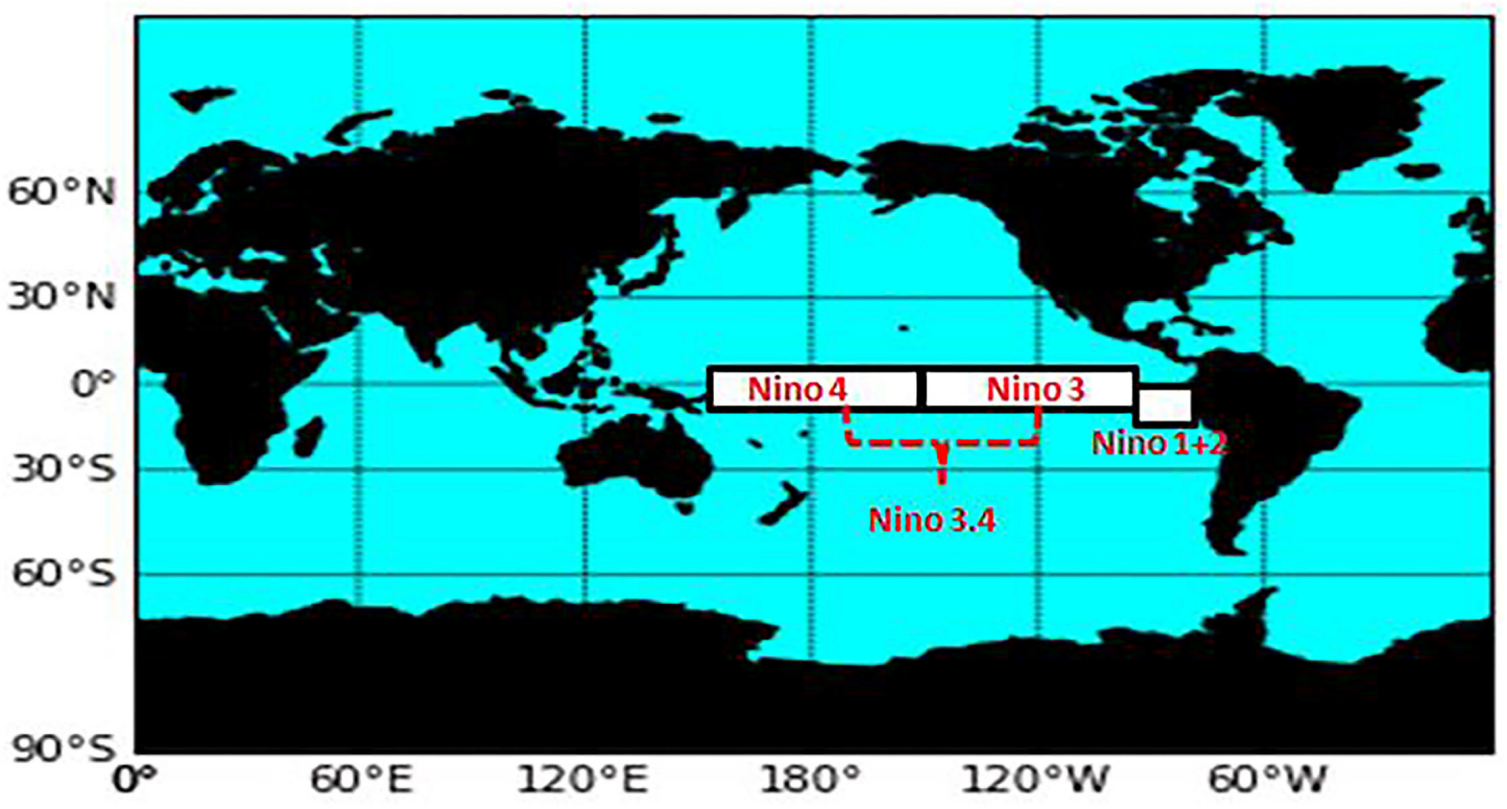
Niño SST indices regions.

Changes in SST were used to detect the status of the El Niño –Southern Oscillation (ENSO) (58). ENSO is said to affect not just weather events but also public health worldwide (59). This paper used the Niño SST indices as one of the key variables in the model together with the Stringency Index (SI) for predicting COVID-19 cases in South Korea. Figure 2 shows the approximate location of the Niño SSTs.

### Stringency Index

The data were collected from the publicly available database (www.ourworldindata.org) maintained by the Oxford Coronavirus Government Response Tracker (OxCGRT). The group used the following formula in calculating the indices:


(1) $$\begin{array}{r} \text{Stringency Index}=\;\frac{1}{k}\;\overset{k}{\underset{j=1}{\sum }}I_{j} \end{array}$$

where k corresponds to the number of component indicators in each index, j as the indicator, and I as the sub-index score. This paper did not calculate the Stringency Index (SI) of South Korea but used the SI values calculated by the Oxford Coronavirus Government Response Tracker (OxCGRT).

The non-pharmaceutical intervention represented by government restrictions (e.g., social distancing, lockdowns, quarantines) is represented by SI. SI is a measure of response metrics in terms of school closures, workplace closures, cancellation of public events, restrictions on public gatherings, closures of public transport, stay-at-home requirements, public information campaigns, restrictions on internal movements, and international travel controls (60). This study used SI to explore the behavior of COVID-19 cases about the change in the restrictions in South Korea for the year 2020.

### Convergent Cross-Mapping

Finding causal relationships and interactions among variables in complex systems is a very valuable aspect of evidenced-based studies such as those concerning disease prevention and public health (61). Causation can imply a correlation, but correlation does not necessarily imply causation. Therefore, this paper used Convergent Cross-Mapping (CCM) to show the effects of SIs and Niño SST indices on the COVID-19 cases in South Korea. Another approach for detecting causality is Granger Causality, which, as emphasized by Sugihara et al. (62), is more applicable to stochastic and linear systems. Granger Causality (GC) is a type of causality test by Granger (63) but is more suitable for stochastic and linear systems. As pointed out by Sugihara et al. (62), CCM can cater to elements where GC is not valid (e.g., non-separable systems or systems where the predictability of some variable Y is not independently unique to another variable in consideration). Thus, CCM is a more suitable approach for dynamic systems and can also distinguish interactions among systems from shared variables.

As discussed in his paper, CCM can test causation for a dynamic system that is not entirely random and can distinguish the correspondence between states, and the longer the time series length, *L*, the more precise the CCM could estimate. Sugihara et al. (62) explained this scenario in their paper by presenting the Lorenz system with two shadow manifolds (or low dimensional representation of the entire system) *M*_*x*_ and *M*_*y*_ constructed using lagged-coordinate embedding (τ = *lag*). It was mentioned that due to the increased library size or time series length, *L*, the shadow manifolds will be much denser, which causes a more precise estimate. τ is simply the lag time, which can be randomly assigned.

**Figure 3:**
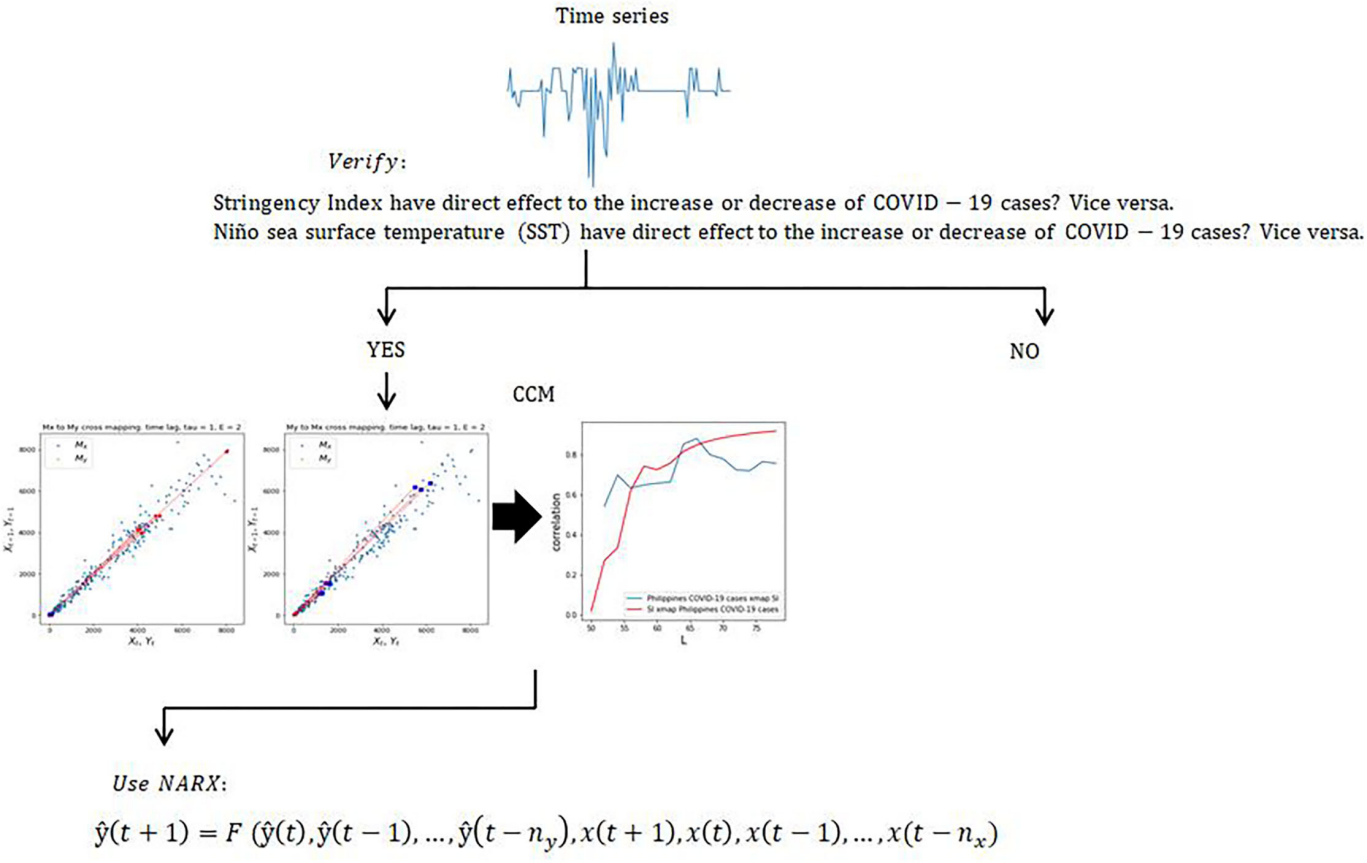
Schematic diagram of the methodology.

In this study, we used CCM to correlate COVID-19 cases, SST, and SI using their respective time series data. Equation (1) and (2) represents the manifolds after the application of CCM where τ = *lag*. Figure 3, on the other hand, shows the schematic diagram of the methodology used in this study.


(2) $$\begin{array}{rcl} M_{x}:\;x\left(t\right) & = & \left[x\left(t\right),\;x\left(t-\tau \right),\;x\left(t-2\tau \right)\right] \end{array}$$

(3) $$\begin{array}{rcl} M_{y}:\;y\left(t\right) & = & \left[y\left(t\right),\;y\left(t-\tau \right),\;y\left(t-2\tau \right)\right] \end{array}$$

As explained by Sugihara et al. (62), we can denote *m(t)* as a point in the manifold, *M*, and *X* an observation function in some temporal flow. For each function, *X*, there is a corresponding time-series, in which we can denote as *{X}* = *{X(1)……X(L)}* that can track the trajectory of the points within the manifold, M (the length of time series or library size is denoted as *L*).

On the other hand, using *x(t)* as points in the manifold, say *M*_*x*_ (which was constructed through the use of the time lagged values of *X*), and which *x(t)* consists of vectors such as *X(t), X(t-* τ*), X(T-2* τ*)* up to *X(t-(E-1)* τ) with *E* as the dimensional state space and τ as the lag time, we can say that *x(t)* on *M*_*x*_ can map *m(t)* on *M*. Thus, in case of two dynamically coupled variables, say *X* and *Y*, their manifolds *M*_*x*_ and *M*_*y*_ can eventually map each other since they are a diffeomorphic reconstruction of their common manifold, *M*. Therefore, it should be expected the presence of an increasing correlation as *L*, or the library size increases. In this paper, we used the *causal-ccm package* in python (64) for CCM analysis.

### Non-linear Autoregressive Exogenous Model (NARX)

The main objective of this study was to develop a case prediction model for COVID-19 using the NARX model with the novel combination of Niño SST (climate variable) and SI (policy index) as variables. This research aims to provide initial evidence that COVID-19 cases may be influenced by climatic factors such as Nino indices and non-pharmaceutical interventions as represented by SI. Several studies (48, 51, 65) have used NARX for disease prediction which proved NARX's promising ability. NARX is a type of neural architecture modeling tool (51), which makes use of the past values of a time series alongside the current and past values of the exogenous variables, which contribute to the time series of interest. As discussed by (66), NARX has two different model architectures, namely: (56) series-parallel or open-loop architecture; and (1) parallel architecture or close loop architecture.

In the paper of Akhtar et al. (51), he used series-parallel NARX to predict the real-time risk of Zika virus in the Americas. Boussaada et al. (66), on the other hand, used the series-parallel architecture of NARX only for the training phase, while the parallel architecture was used in the testing and prediction phase. This study utilized the parallel architecture, which is represented by Equation 4 for the model prediction phase.


(4) $$\begin{array}{l} y\left(t+N\right)=F\;(y\left(t\right),\;y\left(t-1\right),\;\ldots ,\;y\left(t-n_{y}\right),\;x\left(t+1\right),\;\\ \;x\left(t\right),x\left(t-1\right),\ldots ,x\left(t-n_{x}\right) \end{array}$$

NARX model is a type of model structure, which can predict the parent series of *y*(*t*), given the past values of the same time series and another time series, say *x(t)* (48). *y*(*t*+*N*) is the number of cases at time, *t*, and days ahead *(N)*. *x*(*t*) is the independent variable input (Niño SST index as well as Stringency Index (SI) at time, *t*, while *x*(*t*−*n*_*x*_) is the independent variable input at *n* days before time *t*. In this study, we used the *fireTS package* in python to predict the number of cases in South Korea using the data available from January 21, 2020 to September 30, 2020, as the training data *(n* = *254)* and the October 1, 2020 to December 31, 2020, as the testing data *(n* = *92)*. The non-linear mapping function *F(.)*, where a number of nodes, number of layers, or simply the mathematical expression, which transforms the data is approximated by multilayer perceptron (MLP) during the training process and is, therefore, unknown. MLPs are used to approximate continuous functions and are designed to solve complex non-linear separable systems (67).

### Performance Indicators Employed

Root mean square error (RMSE), Nash-Sutcliffe efficiency (NSE), an index of agreement (IA) were utilized as metrics for evaluation. RMSE is obtained from the difference between simulated and actual values, therefore, it is an indicator of how much error the simulated results contain vs. the actual value. NSE is an indicator that shows how well the plot of predicted and observed data fit on a 1:1 line. This metric has values that range from 0 to 1. An NSE = 1 indicates a perfect agreement between the predicted and the observed values, while NSE = 0 means the predicted is as accurate as of the observed data. On the other hand, an NSE < 0 means the observed data is a better predictor compared to the predicted values.

Willmott (68) defines the index of agreement (IA) as the ratio of the mean square error (MSE) and the potential error (PE) multiplied by the number of observations. This value is then subtracted from one. IA values range from 0 to 1 with higher index values suggesting better agreement between the observed and simulated values.

## Results

Detecting causation from the time series using CCM of South Korea COVID-19 cases and El Niño SST indices are shown in Supplementary Figure 1. The downward trend (concerning the length of the dataset being analyzed) of the black curve signify that the COVID-19 cases did not influence the Niño SST indices, while the increasing and stable green curve show that Niño SST indices had an influence on the COVID-19 cases. For the Niño SST indices influencing COVID-19 cases (green curves), the correlation values reached high levels (ρ ≈ 0.70 for Niño 3.4; ρ ≈ 0.71 for Niño 3; ρ ≈ 0.71 for Niño 4; ρ ≈ 0.74 for Niño 1+2). This means that among the Niño SST indices, Niño 1+2 was the most influential in COVID-19 cases in South Korea.

When the CCM of SI and the COVID-19 cases were plotted (Supplementary Figure 2), an increasing and decreasing trend for the COVID-19 cases influences SI (black curve) were found while the SI influence COVID-19 cases (green curve) had an increasing trend followed by a much stable trend was plotted. The correlation values of SI influence to South Korea COVID-19 cases (green curves) reached as high as 0.86 with around 0.80 as the highest correlation for the the COVID-19 cases influence SI (black curve).

**Figure 4:**
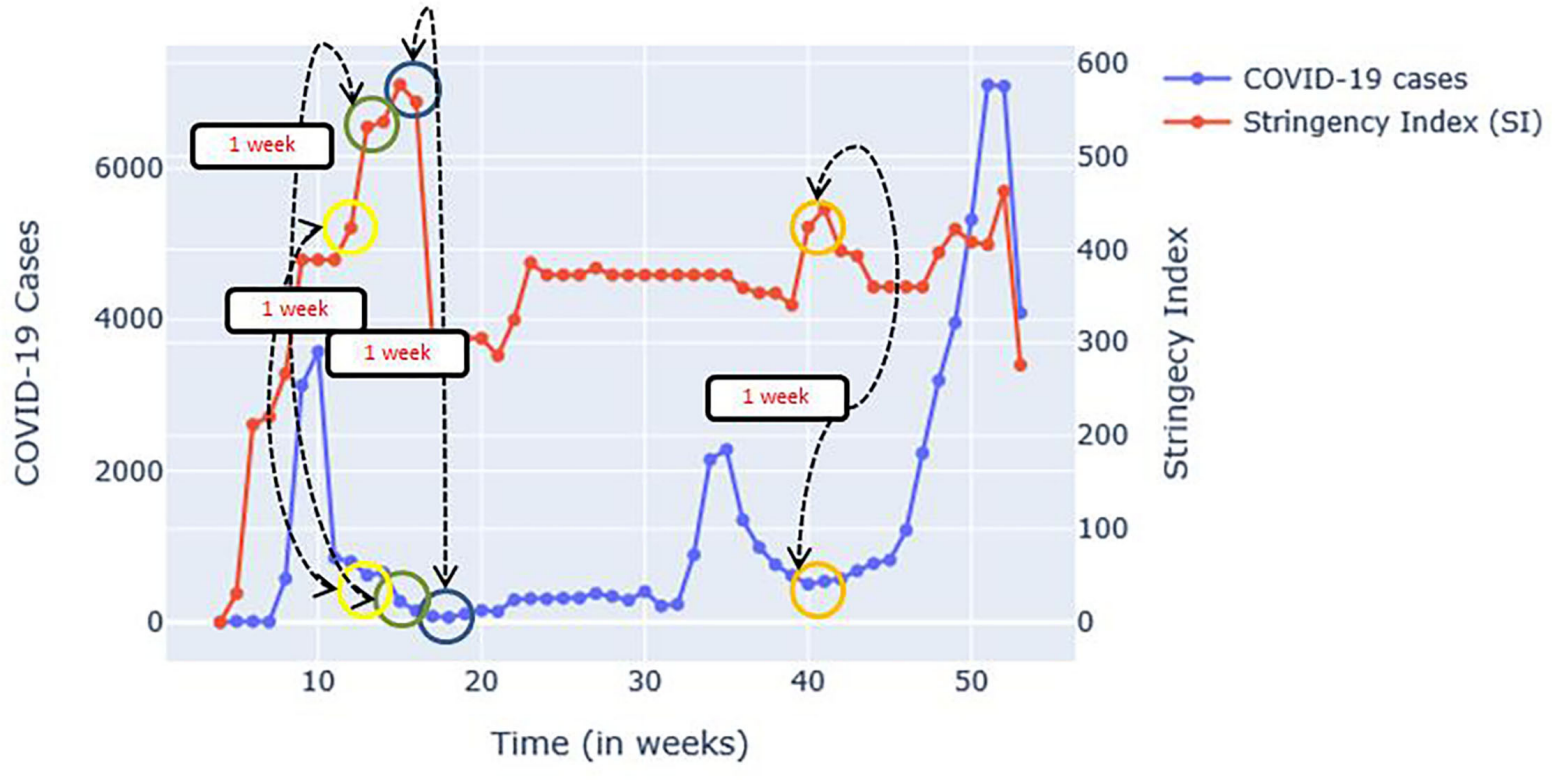
The plot of SI and COVID-19 cases.

**Figure 5:**
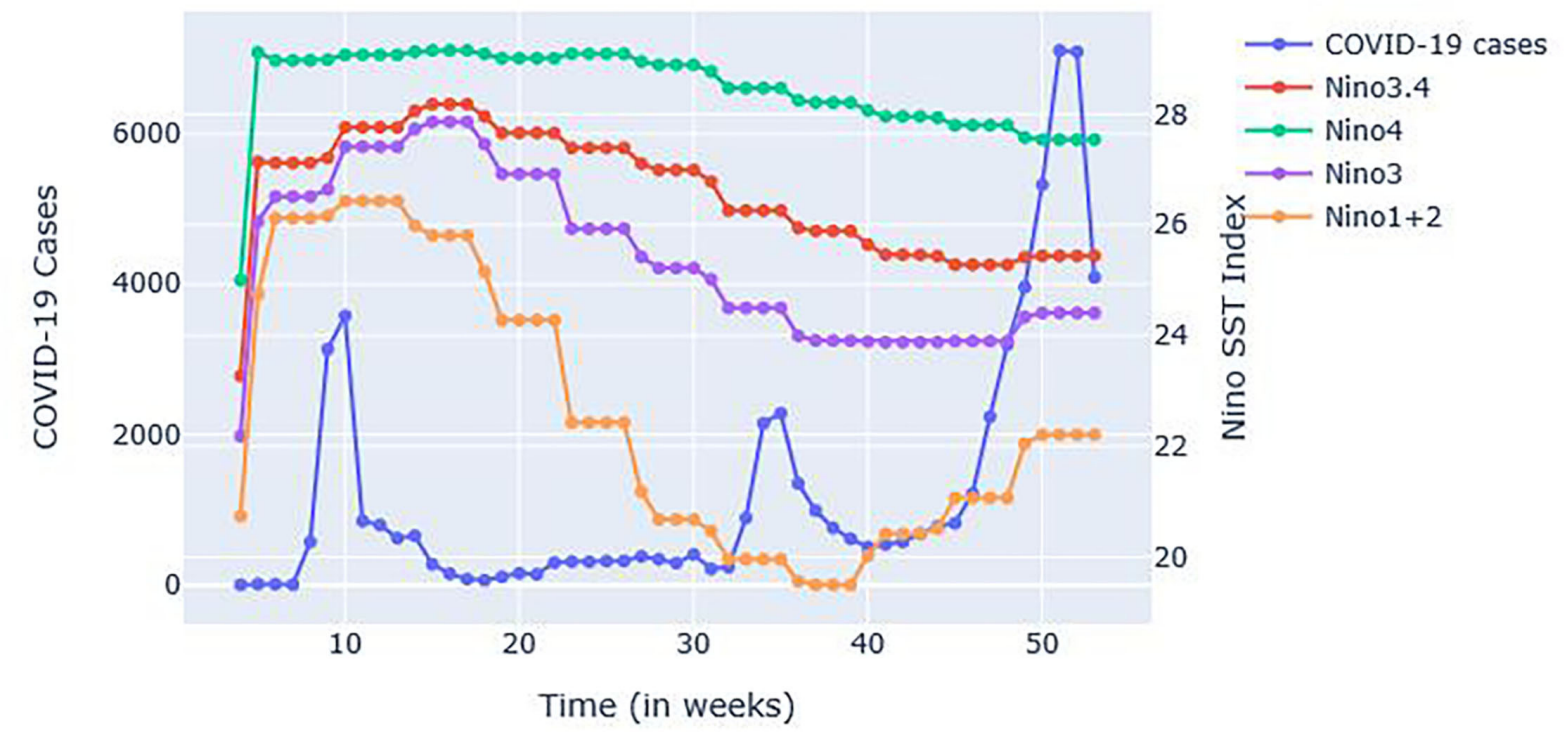
Plot of Niño SST Indices and COVID-19 cases.

Figure 4 shows a plot of COVID-19 cases and SI plotted simultaneously. It is worth noting that at points where SIs are high [See week 15 (April), 40 (October), and 41 (October) most especially], the number of COVID-19 cases is very low a week after the implementation. The simultaneous plot of SST cases and COVID-19 in Figure 5 have shown that in Weeks 35 (September) onwards, where the trend of COVID-19 cases has decreased (Weeks 35–39 or September-October) and increased (Weeks 40 onwards or October onwards), the same trend has been observed to Niño1+2 index which obtained the highest correlation value based on the CCM analysis. Other Niño SST indices showed almost the same trend but the decrease and increase were lesser than Niño 1+2. In Weeks 4–10 (January-March), an increasing trend was found for the Niño SSTs [an increase of around 5.68, Week 4 (January) at 20.75 and Week 10 (March) at 26.43 for NIÑO 1+2] and the number of COVID-19 cases (which ranges from 2 to 3,578), while another decreasing trend was observed in Weeks 10–20 (March-May) for both COVID-19 cases [which ranged from 848 for Week 11 (March) to 156 for Week 20 (May)] and Niño SSTs, with Niño 1+2 [which ranged from 26.43 for Week 11 (March) to 24.28 for Week 20 (May)] showing a much steeper decrease in values compared to other Niño SST indices [Niño 3.4 ranged from 27.76 for Week 11 (March) to 27.66 for Week 20 (May); Niño 4 ranged from 29.07 for Week 11 (March) to 29.01 for Week 20 (May); Niño 3 ranged from 27.41 for Week 11 (March) to 26.92 for Week 20 (May)].

It is also worth noting that CCM alone cannot quantify how much SI caused a decrease or an increase in the COVID-19 cases as it only shows if one time series has an influence on the increase of COVID-19 cases and is supposed to exhibit an increasing correlation. Upon expanding the length of the time series (L), and as shown in Supplementary Figure 2, the correlation values of the SI influencing COVID-19 cases (green curves) reached as high as 0.86. To provide clarity, the SI influencing COVID-19 cases (green curves), which reached as high as 0.86 and became stable [or reached a “plateau” as termed by Sugihara et al. (62)], is an estimated precision (or correlation), where the manifolds, say M_x_ (SI) will converge to another manifold, say M_y_ (Cases).

We incorporated both Niño SST indices and SI to the NARX model using January 21, 2020 to September 30, 2020, as the training data and October 1, 2020 to December 31, 2020, as the testing data. The performance of each model in varying Niño SST index was then measured using RMSE, NSE an,d IA. The values of the performance indicators can be seen in **Table 2**.

The values of the IA were all within a satisfactory limit (0.86–0.88) as shown in Table 2. The same goes with the NSE, which ranged from 0.66 to 0.69. The RMSE values were low compared to the average number of COVID-19 cases, which, by convention, means a better prediction model since the average a number of COVID-19 cases was more than 400 [from October 1, 202,0 to December 31, 2020, (see Table 1B)].

**Table 1B:** Descriptive statistics of the data used (October 1, 2020 to December 31, 2020).

	**SI**	**Cases**	**Niño3.4**	**Niño4**	**Niño3**	**Niño1+2**
Count	92	92	92	92	92	92
Mean	57.47	411.74	25.39	27.77	24.07	21.24
Std	5.44	370.25	0.08	0.17	0.25	0.75
Min	51.39	47	25.28	27.54	23.88	20.42
Max	68.98	1,237	25.46	27.96	24.41	22.21

Supplementary Figure 3 shows that x = COVID-19 cases and y = Niño SST indices and SI; M_x_ and M_y_ manifolds showed that local neighborhoods on M_y_ correspond to the local neighborhood on M_x_ and vice versa. This can be a support to the high-performance indicators obtained in Table 2.

**Table 2:** Results of performance indicators.

**Y**	**X1**	**X2**	**RMSE**	**NSE**	**IA**
Cases	SI	NIÑO3.4	217.18	0.66	0.86
Cases	SI	NIÑO3	215.68	0.67	0.87
Cases	SI	NIÑO4	216.43	0.67	0.87
Cases	SI	NIÑO1+2	209.50	0.69	0.88

The 3- and 5-day lag predictions are the best fit based on the performance indicators employed are shown in Table 3. However, for practicality purposes, the 7-day lag model which performed well and with performance indicator results not far from the 3- and 5-day prediction (7-day lag RMSE is 206.84, while the 3-day and 5-day have RMSE values of 189.03 and 190.07, respectively (a difference of 17.81 and 16.77). The 7-day lag has an NSE value of 0.72, while both the 3-day and 5-days have an NSEnce value of 0.75 (a difference of 0.03). IA value of the 7-day lag is 0.89, while both the 3-day and 5-days have IA values of 0.91 (a difference of 0.02), which could also be chosen by government officials to implement new policies and regulations concerning COVID-19. The authors believe that the 7-day lag of time prediction (which would include weekends, ideally) would allow the businesses and the citizens, as a whole, to prepare for the needed adjustments due to new or additional stringencies to be imposed by the governments, whether local or national. In the paper of Heo et al. (69), it was d out that the restriction and stringency indices were effective at 10-day lag, closing index after 8-day lag, and health index after 0–5-day lag. However, the SI used in this study by Oxford Coronavirus Government Response Tracker (OxCGRT) is already the combination of all the specific indices. Averaging the results of Heo et al. (69), an estimated 8-day lag can be obtained. In our study, we recommend a 7-day lag for practical purposes. For visualization, the observed and predicted COVID-19 Cases for (a) 3-day; (b) 5-day; and (c) 7-day lag prediction using NARX with SI and Niño 1+2 Index as variables are shown in Supplementary Figure 4.

**Table 3:** Performance of Niño 1+2 N-day prediction model.

	**RMSE**	**NSE**	**IA**
3-Day	189.03	0.75	0.91
5-Day	190.07	0.75	0.91
7-Day	206.84	0.72	0.89
14-Day	262.69	0.58	0.81
21-Day	329.94	0.40	0.71

To check whether the inclusion of Niño SST indices and SIs in our study could potentially improve previously done models, we made another model patterned to the time periods used by recently published articles (data not shown) and found out that the MAPE values have improved. According to Lewis (70), highly accurate forecasting had a MAPE value of <10% which was achieved by incorporating Niño 1+2 and SI into the prediction model.

For the association of COVID-19 cases to SI, a proof can be shown in the paper of Jayaweera et al. (71), when he used a linear regression model to analyze the effectiveness of imposed stringencies (isolation, social distancing, and contract tracing, which were all imposed in South Korea) to mitigate the COVID-19 pandemic, which proved that non-pharmaceutical interventions, such as lockdowns and isolations are effective in averting COVID-19 pandemic.

To check for the relationship between SI and COVID-19 cases, the authors have provided Supplementary Figure 5. In this figure, it was shown that a LOWESS Regression was a better fit for the two variables (linear and logistic regression was found to be inappropriate). LOWESS regression or Locally Weighted Scatterplot Smoothing can create a smooth line to show variable relationships when linear or logistic regressions are deemed inapplicable. Some research about the policy stringency index in Brazil, Mexico, and the United States suggest that SI and COVID-19 cases follow a loess (or lowess) curve (72). On the other hand, Hale et al. (73) used Lowess regression to identify the waves of COVID-19 cases in India, the United States, and South Africa.

## Discussion

Supplementary Figures 1, 2 show the CCM of COVID-19 cases and Niño SST indices, as well as the CCM of COVID-19 cases and SI. As Sugihara et al. (62) emphasized in their paper, there should be an increasing correlation as the time series length or library size (L) increases. The Niño Index influencing South Korea COVID-19 cases (represented by green curves) are much more h stable and constantly increasing as L increases while the South Korea COVID-19 cases influencing Niño Index (represented by black curves) are in a decreasing trend. For the Niño Index influencing South Korea COVID-19 cases (green curves), the correlation values have reached as high as ρ ≈ 0.70 for Niño 3.4; ρ ≈ 0.71 for Niño 3; ρ ≈ 0.71 for Niño 4; ρ ≈ 0.74 for Niño 1+2. This means that among the Niño SST indices, Niño 1+2 was the most influeninal to COVID-19 cases in South Korea.

On the other hand, despite the high correlation values of the South Korea COVID-19 cases influencing the Niño Index (black curves), it did not exhibit a consistently increasing trend as L increased.

In Supplementary Figure 2, the SI had an influence on South Korean COVID-19 cases (represented by green curves) showed an increasing and a more stable trend while the SouthKoreana COVID-19 cases having influence to SI (represented by black curves) also showed an increasing trend at the beginning but decreased subsequently. These findings have become much clearer upon increasing the length of the time series. The results indicate that at the beginning of the 2020 pandemic in South Korea, the implementation of stringencies by the governments might have been influenced by the increase or decreaseinf the reported number of new cases. However, at some point, the influence of occurrence of new cases to the SI lessened (which can be caused by factors, not within the scope of this study, e.g. economic factors, etc). This could mean that COVID-19 cases are influenced by SI but SI is not necessarily being influenced by the number of COVID-19 cases. The correlation values of the SI had an influence on South Korea's COVID-19 cases (green curves) that reached as high as 0.86 with around 0.80 for the South Korean COVID-19 cases that influence SI (black curves).

The numerical patterns in the changes of SI and Niño SST indices are shown in Table 4. As shown, from week number 10–15 (March to April), all the SIs (which were found to have an effect after a week based on Figure 4) are increasing while the cases are decreasing. The same goes to Niño 1+2 SST indices which showed a decreasing trend (Niño 1+2 SST indices at week 15 (April) is 25.81). However, from weeks 39 to 42 (October), the SIs (which again, was found to have an effect after a week based on Figure 4) was found to be increasing from weeks 39 to 41 (October) while it dropped to 56.94 from 63.36 (a drop of 6.42) on week 42 (October). SI of week 40–41 (October) is increasing which affected COVID-19 cases of week 39–40 (October) (decreasing). SIs of week 41–42 (October) has been decreasing, which affected week 40–41 (October) of COVID-19 cases (increasing). Niño 1+2 on the other hand has an increasing trend from week 39 to 41 (October) and the cases from 40 to 42 (October) are also increasing.

**Table 4:** Changes in SI and Niño SST indices patterns.

**Week number**	**SI**	**Cases**	**Niño3.4**	**Niño4**	**Niño3**	**Niño1+2**
10	55.56	3578	27.76	29.07	27.41	26.43
11	55.56	848	27.76	29.07	27.41	26.43
12	60.45	799	27.76	29.07	27.41	26.43
13	75.93	622	27.76	29.07	27.41	26.43
14	76.72	654	28.06	29.13	27.73	25.99
22	46.36	297	27.66	29.01	26.92	24.28
23	55.09	311	27.39	29.09	25.93	22.43
24	53.24	307	27.39	29.09	25.93	22.43
39	48.61	616	25.89	28.21	23.91	19.50
40	60.59	503	25.64	28.07	23.89	20.03
41	63.36	539	25.46	27.96	23.88	20.42
42	56.94	572	25.46	27.96	23.88	20.42

However, in week 22–23 (June) where a decrease in Niño 1+2 SST indices was recorded (24.28 and 22.43) a slight decrease of COVID-19 cases was also found on week 23–24 (June) (from 311 to 307) despite the decrease of SI from week 23–24 (June) (from 55.09 to 53.24). In week 22–23 (June), the SIs were increased (from 46.36 to 55.09) but the cases from week 21–22 (May to June) increased too (see Figure 4).

The graphical representations (Figures 4, 5) show that the two variables (Niño SST indices and SIS) have effects on the increasing or decreasing trend of COVID-19 cases at least a week after the increase or decrease of SIs and Niño SSTs. Although factors such as number of tests done could play a huge role, the patterns mentioned could mean that Niño SSTs can also be used as one of the factors by governments in deciding for the appropriate restrictions to implement (e.g., if the Niño SSTs which is seasonal is already on its increasing pattern, the SI to be implemented should also be increasing to avoid increasing number of cases).

In the paper of Oluwole (74), he emphasized that El Niño Southern Oscillation (ENSO) determines the timing and severity of influenza epidemics therefore influencing the seasonality of diseases. According to WHO (75) (https://www.who.int/emergencies/diseases/novel-coronavirus-2019/question-and-answers-hub/coronavirus-disease-COVID-19-similarities-and-differences-with-influenza), COVID-19 and influenza are respiratory diseases sharing many similar symptoms although the causative viruses and treatment may be different. Thus, possible seasonality of COVID-19 cases (just like influenza) can be associated to Niño SST indices which are affected by ENSO.

Kolle et al. (76) and Ma et al. (77) reported that an increase in physical activity appears in the spring season in Norway and USA. In South Korea, increased COVID-19 cases were observed in the months of February to March then September, which are during the spring and autumn season. Cayan (78) emphasized the relationship of SST and surface air temperature (SAT). In his paper, he proved the contemporaneous correlation between the two. If SAT is hot, it means that air molecules are far from each other and, therefore, less dense. Cold air is more dense due to closer molecules and reduced movement, which can contribute to disease transmission. Menebo (29) observed also a positive association between the daily COVID-19 cases with the maximum and normal air temperature.

The use of the NARX model in predicting the number of potential COVID-19 cases, which could arise using SI and SST indices as variables have been proven to be effective due to the low RMSE and high NSE and IA values, which all signify that the model was able to represent accurately the actual COVID-19 cases in South Korea. Due to the inclusion of Niño SST indices and SIs in our study, the MAPE values have improved, which implies that the combined variables of SST and SI when used with NARX (especially using SI and NIÑO 1+2), which produced a MAPE value of 0.27 is effective in predicting the number of COVID-19 cases.

Despite the satisfactory results presented, one of the limitations in this paper is that the modeling approach was limited to only two variables and other potential confounding variables were not included. It could be that the observed impact of the Niño SST variable on transmission of SARS-CoV-2 which we observed might be secondary and its effect might be of a lesser degree compared to the impact of other factors such as mobility, non-pharmaceutical or policy interventions (79, 80). Another limitation is that the research is limited to a 1-year (2020) data, therefore effects due to vaccination or the introduction of SARS-CoV-2 variants associated with significantly higher transmission rates (i.e., Delta) were not considered. For future studies, prediction models considering vaccination data encompassing 2021 (and onwards) could be done using the same modeling approach but with incorporation of another variable such as vaccination index. Vaccination indices should cover the geographical data and the number of vaccinations (1st, 2nd, 3rd, or 4th booster shots) administered/received as well as the age of the recipient. In countries where the vaccination of COVID-19 is not fully funded by the government, donations or those procured *via* the COVAX facility should also be included as indicators of vaccination indices. The authors would also like to emphasize that the specific measure for each SI is beyond the scope of this study. Based on OxCGRT, these specific measures are divided into five categories namely Containment (C), Economic (E), Health (H), Vaccination (V), and Miscellaneous (M) policies which are subdivided further. Thus, for future studies, a more specific SI calculation encompassing the sub-indices would be recommended in order to know specific regulations to be implemented for each change in environmental variables such as Niño SST indices.

Another limitation is that this paper did not consider biological and behavioral factors, such as reduced or heightened or human physical activity (81) or mobility. The socioeconomic background (which can affect a country's healthcare capabilities) was also not considered.

## Conclusion

In summary, the study shows that the use of NARX with novel combination of Niño SST (climate variable) and SI (policy index) as variables could be used to predict COVID-19 cases. Accuracy of SARS-CoV-2 transmission forecasts across seasons may be further improved by incorporating both the Niño SST and SI variables and combining these variables with NARX, may outperform other models. This study found that the optimum number of days the government can impose stringencies range from 3 to 7 days with preference to 7 days to accommodate adjustments which need to be done by the businesses and other essential establishments. Findings of this study will enable policymakers not just to monitor the increase or decrease of cases but also enable them to make timely announcements, interventions and impose restrictions in order to avoid high hospital bed and intensive care unit (ICU) utilization rates in hospitals. Niño 1+2 SST indices were also found to be the most influential on COVID-19 cases based on the CCM.

As SARS-CoV-2 potentially evolves into an endemic pathogen, this study provides initial evidence that COVID-19 cases may be influenced by climatic factors, such as Nino indices and public health, and non-pharmaceutical interventions can be timed based on the identified factors. Our findings could help push for more climate- or environmental-friendly policies, which could help slow down global warming which causes the increase in the warming of sea surface temperatures. Future forecasting work by modelers should consider including climate or environmental variables (i.e., Niño SST) to enhance prediction of transmission and spread of SARS-CoV-2. In order to further improve the accuracy of the predictions generated by our model, we recommend the incorporation of additional meteorological and vaccination variables, behavioral and biological factors as well as country-specific socio-economic capacity.

## Data Availability Statement

The original contributions presented in the study are included in the article/Supplementary Material, further inquiries can be directed to the corresponding author.

## Author Contributions

IN: conceptualization, formal analysis, investigation, methodology, and writing—original draft. JV: conceptualization, supervision, and writing—review and editing. JJ, YB, and YY: writing—review and editing. SK and HK: supervision and writing—review and editing. All authors contributed to the article and approved the submitted version.

## Funding

This work was supported by the National Research Foundation of Korea (NRF) grant funded by the Korea Government (MSIT) (No. 2017R1A2B3005695).

## Conflict of Interest

The authors declare that the research was conducted in the absence of any commercial or financial relationships that could be construed as a potential conflict of interest.

## Publisher's Note

All claims expressed in this article are solely those of the authors and do not necessarily represent those of their affiliated organizations, or those of the publisher, the editors and the reviewers. Any product that may be evaluated in this article, or claim that may be made by its manufacturer, is not guaranteed or endorsed by the publisher.
