# Supplementary figures and images for: Predicting COVID-19 Cases in South Korea Using Stringency and Niño Sea Surface Temperature Indices
Source: Front Public Health. 2022 Jun 3;10:871354. doi: 10.3389/fpubh.2022.871354 (PMC9204014; doi:10.3389/fpubh.2022.871354)

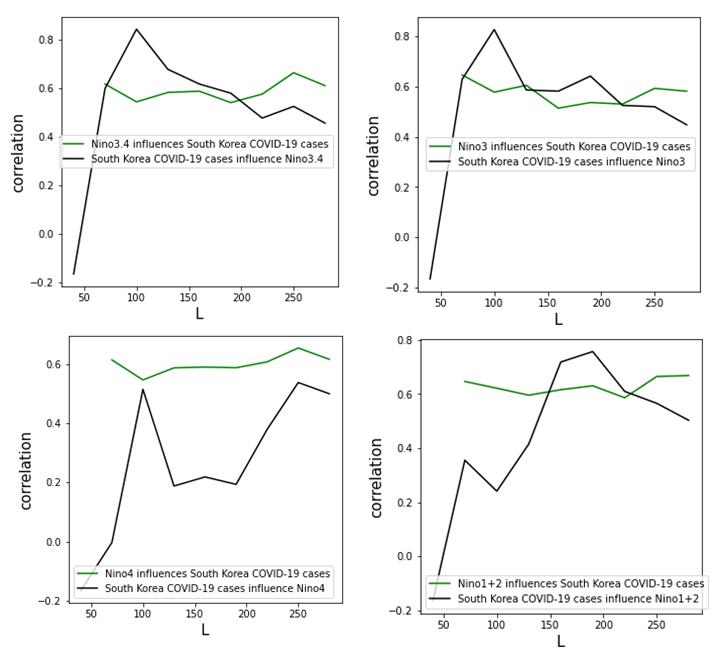

Supplement: Supplementary file 1 [file Image_1.JPEG]

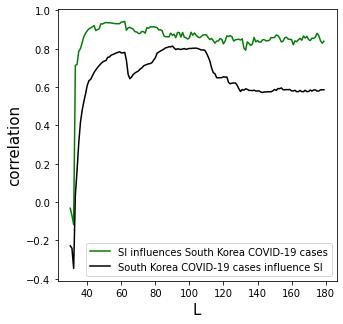

Supplement: Supplementary file 2 [file Image_2.JPEG]

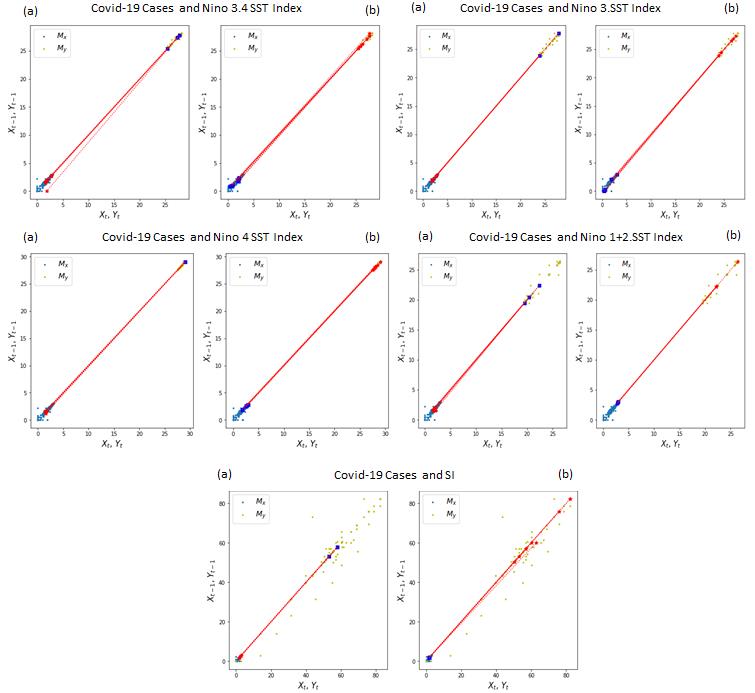

Supplement: Supplementary file 3 [file Image_3.JPEG]

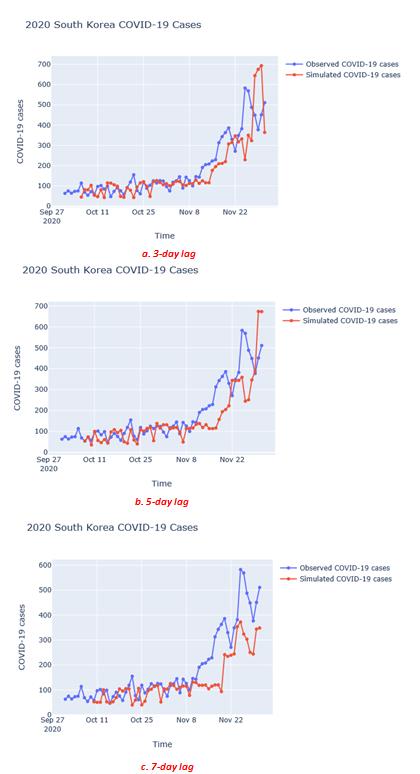

Supplement: Supplementary file 4 [file Image_4.JPEG]

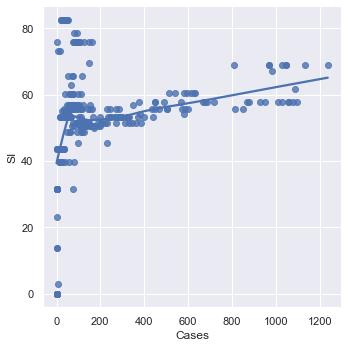

Supplement: Supplementary file 5 [file Image_5.JPEG]
